# Genomic analysis of a Palestinian family with inherited cancer syndrome: a next-generation sequencing study

**DOI:** 10.3389/fgene.2023.1230241

**Published:** 2023-10-31

**Authors:** Eman Fares, Rua Thawabtah, Husam Sallam, Areej A. H. Khatib, Nouar Qutob, Zaidoun Salah

**Affiliations:** ^1^ Health Sciences Department, Faculty of Graduate Studies, Arab American University, Ramallah, Palestine; ^2^ Women Health Research Unit, McGill University Health Center, Montreal, QC, Canada

**Keywords:** cutaneous melanoma, xeroderma pigmentosum, NER, Palestinian family, ERCC2/XPD, cancer syndrome

## Abstract

Familial predisposition is a strong risk factor for different types of cancer and accounts for around 10% of the cases. In this study, we investigated cancer predisposition in a Palestinian family using whole-exome sequencing (WES) technologies. In this study, we focused more on cutaneous melanoma (CM). Our analysis identified three heterozygous rare missense variants, WRN (p.L383F and p.A995T) and TYRP1 (p.T262M) and a pathogenic homozygous missense mutation in ERCC2 (p.R683Q). Although WRN and TYRP1 genes and their variations were correlated with different types of cancer, including melanoma, the currently identified WRN and TYRP1 variants were not reported previously in melanoma cases. The pathogenic mutation was segregated with the clinical phenotypes and found in the two affected brothers, one with CM and the other with brain tumor, and was confirmed by Sanger sequencing analysis. Segregation analysis of this mutation revealed that family members are either heterozygous or wild type. Our findings confirm that the homozygous ERCC2 (p.R683Q) mutation was responsible for causing melanoma and other cancer types in the family. Our work highlights the value to decipher the mutational background of familial cancers, especially CM, in the Palestinian population to guide diagnosis, prevention, and treatment of affected patients and their families.

## Introduction

The incidence of melanoma has steadily increased over the last few years (Ueda, 2000). In Palestine, according to the Globocan statistics 2020, the incidence rate of skin melanoma was 0.46%, ranking 26 among cancer types (The Global Cancer Observator, 2021). It is worth noting, however, that cancer incidence in Palestine is affected by incomplete reporting and surveillance issues (Halahleh and Gale, 2018). The pathogenesis of CM (cutaneous melanoma) is complex, with environmental and genetic factors affecting disease risk (Schadendorf et al., 2018). Most melanoma cases are sporadic and caused by acquired mutations occurring in somatic cells (Society, 2016). In sporadic melanoma, somatic mutations generally induce activation of the MAPK signaling pathway, most commonly in BRAF or NRAS (Sankar, 2020). Around 10% of CM cases are familial, occurring in individuals with two or more affected first-degree relatives (Goldstein et al., 2007; Ransohoff et al., 2016). Moderate-to high-penetrance genes (*CDKN2A, CDK4, BAP1, TERT, POT1*, *ACD, MITF, MC1R,* and *TERF2I*) have been shown to effect predisposition to melanoma and account for approximately 50% of familial cases (Goldstein et al., 2008; Puntervoll et al., 2013; Aoude et al., 2014; Robles-espinoza et al., 2014; Harland et al., 2016). Therefore, the etiology of the remaining familial cases is largely unknown, suggesting the presence of rare high-penetrance genes that are likely to exist or a combination of multiple low-penetrance genes and/or their interaction with environmental risk factors. Genome-wide association studies (GWAS) have identified more than 20 low-risk variants linked to melanoma susceptibility. Most of these are found in genes involved in multiple pathways that are known to play critical roles in melanoma development, such as pigmentation (*TYR, TYRP1, ASIP,* and *HERC2/OCA2*), nevi density (*PLA2G6, MTAP/CDKN2A, CASP8, AGR3*, and *FTO*), DNA repair (*XRCC3, XPD, ATM*, and *PARP1*), and metabolism and detoxification (*GSTM1* and *GSTT1*) (Fargnoli et al., 2006; Goldstein et al., 2017; Artomov et al., 2018). In addition to melanoma susceptibility genes, an increased risk of melanoma has been reported in patients with germline mutations causing cancer syndromes, including hereditary breast and ovarian cancer (BRCA1/BRCA2), Werner syndrome (WRN), and xeroderma pigmentosum (XP) (Ransohoff et al., 2016). The ERCC2 gene plays critical roles in transcription and DNA repair, and mutations in this gene lead to the development of diseases such as XP and TTD (Coin et al., 1998). Moreover, mutations in ERCC2 were connected with different types of cancer, including melanoma (Manuguerra et al., 2006; Dong et al., 2013). Advancement in next-generation sequencing technology has enabled the complete characterization of a large number of genes to identify alterations underlying melanoma development, leading to a better management and treatment strategy. The main objective of this study was to investigate the genetic cause of melanoma in a Palestinian family using the WES approach.

## Materials and methods

### Clinical phenotyping and family pedigree

Seven members of a Palestinian family, of which one individual is affected with melanoma (9), two with a brain tumor (3 and 6), and four unaffected individuals (2, 4, 5, and 10) were involved in the study. A family history of melanoma emerged, with two siblings diagnosed with melanoma at the ages of 29 and 17. The patient’s father and two paternal uncles were diagnosed with prostate cancer. The clinical phenotypes of the patient (9) and individual (6), who had a brain tumor with no melanoma, were abnormality of skin pigmentation and hyperpigmentation of the skin. Individual (5) had normal skin pigmentation. No clinical data were available for the rest of the family members included in the study (Table 1).

**TABLE 1:** Demographic data and clinical phenotypes of the Palestinian family with melanoma. (-): not present.

Family members (ID)	Relationship with patient (II 9)	Age (years)	Sex	Clinical phenotype
II 9	-	30	Male	Skin melanoma, abnormality of skin pigmentation, and hyperpigmentation.
II 5	Brother	-	Male	Normal pigmentation
II 6	Brother	36	Male	Brain tumor, abnormality of skin pigmentation, and hyperpigmentation.
II 7	Brother	29	Male	Skin melanoma
II 8	Brother	17	Male	Skin melanoma
II 1	Sister	-	Female	-
II 2	Sister	-	Female	-
II 3	Sister	33	Female	Brain tumor
II 4	Brother	-		-
II 10	Wife	-	Female	-
I 1	Father	-	Male	Prostate cancer
I 3	Paternal uncle	-	Male	Prostate cancer
I 4	Paternal uncle	-	Male	Prostate cancer
III 1	Child	-	Female	-

### Sample collection

Approximately 5 ml of whole blood was collected from the participating subjects in EDTA tubes. All subjects signed a consent form expressing their willingness to participate in this study. All methods were performed in accordance with the relevant guidelines and regulations of the AAUP ethical committee.

### Whole-exome sequencing

DNA was extracted using the Promega Blood kit, catalog #A1120 according to the manufacturer’s instructions. DNA concentrations and purity were assessed using the Thermo Scientific NanoDrop 2000c. WES was performed on the DNA of the patient and the two unaffected siblings, one of them previously diagnosed with a brain tumor. DNA libraries were generated using the Illumina DNA Prep with Enrichment—(S) Tagmentation, 16 Samples kit, catalog # 20025523, following the manufacturer’s instructions. Library yield was determined using the Qubit dsDNA HS Assay Kit, catalog # Q32850, and the mean fragment size was determined using an Agilent Technology 2100 Bioanalyzer with a High-Sensitivity DNA kit, catalog #5067-4626. Denaturation and final loading concentration of the libraries were performed according to the Denature and Dilute Libraries Guide for NextSeq 500 and NextSeq 550 Sequencing Systems. The prepared libraries were sequenced on a NextSeq 550 platform with 40 X depth. VCF files with annotations of variant effects were created using the SnpEff tool, which annotates variants with their computed impacts on known genomic characteristics. Secondary analysis was conducted using DRAGEN pipeline (Illumina).

### WES variant prioritization

The output variant call format (VCF) file contained 36,521 variants and was filtered based on several filtering criteria, as follows. First, variants that were intronic, ncRNA_intronic, downstream, upstream, 3’UTR3, 5’UTR, and synonymous unknown were filtered out. Then, variants were assessed by their clinical significance from the ClinVar database. They were excluded if they have been reported as benign and likely_benign. Next, variants were filtered out if they had a general allele frequency (AF) and Greater Middle Eastern allele frequency (GME_AF) above 0.1%, based on the gnomAD database. This filter ensures that the non-filtered variants have a level of evidence for pathogenicity and are not polymorphisms. In order to limit false positives, variants with mapping by quality (MQ) scores below 60, which are low-quality scores that have a higher probability of error, were all removed. In addition, variants having quality by depth (QD) scores below 3 were removed. Then, variants were filtered out based on the exomiser, which ranks candidate variants according to the patient phenotype similarity to known disease–gene phenotypes, using the human phenotype ontology terms (HPO). Variant scores were scaled from 0 to 1, and scores lower than 0.5 were filtered out. Following exomiser filtering, the genotype of the patient was evaluated, removing variants with wild-type alleles (WT), and considered either homozygous (HOM), heterozygous (HET), or low allelic balance (lowAB) genotypes. Next, all variants that are unrelated to cancer or melanoma according to OMIM and ClinVar databases were removed. After that, synonymous and ncRNA_exonic variants were filtered out. Finally, only variants that were present in both the patient and his brother with a brain tumor, but not in the control, were kept. After filtering, four variants in three genes remained for further analysis.

### Variant selection

Candidate variants shared between the patient (9) and his brother (6) diagnosed with a brain tumor were further filtered for Sanger sequencing. Only variants that are reported as pathogenic or potentially pathogenic with a genotype–phenotype association based on ClinVar were selected.

### Sanger sequencing

The identified homozygous mutation in ERCC2, p.R683Q, was confirmed by Sanger sequencing, and co-segregation analysis was performed on family members II 2, II 3, II 4, and II 10. For Sanger sequencing, polymerase chain reaction (PCR) was utilized to amplify the targeted regions using the following primers: forward primers: tca​ggt​tga​ggt​tgg​cat​ct; reverse caa​gaa​cca​ggc​tgt​ttc​cc. After purification of the PCR products, Sanger sequencing was run using the BigDye Terminator v3 kit (Applied Biosystem) according to the manufacturer’s instructions. The sequences of the PCR products were viewed and analyzed against the human reference genome hg19 (UCSC Genome Browser).

### *In silico* analysis and interpretation of the candidate gene

*In silico* analysis was performed to predict the functional consequences of the validated variant using several tools: SIFT, PolyPhen-2, PROVEAN, MutationTaster, and Align GVGD. The protein domains of the candidate gene were studied to locate the variant. To check the evolutionary conservation of the sites of the variants, the amino acid sequences of the candidate gene from *Homo sapiens* and other species were gathered from the NCBI and aligned with COBALT (https://www.ncbi.nlm.nih.gov/tools/cobalt/cobalt.cgi). Moreover, 3D structural modeling was performed to determine the site of the mutation using the Protein Data Bank (PDB) database. The candidate variants were interpreted and classified according to the ACMG guidelines. After that, the somatic mutation databases, Catalogue of Somatic Mutations in Cancer (COSMIC), and The Cancer Genome Atlas (TCGA) cBioPortal for cancer genomics were accessed to check whether the candidate variant is present in tumors of melanoma and other cancer types. The *in silico* tool, Phyre2 (http://www.sbg.bio.ic.ac.uk/phyre2/html/page.cgi?id=index) was used to predict the protein structure based on the homology modeling technique. On the other hand, the candidate gene was investigated for alternation frequency in melanoma and other cancer types using the TCGA cBioPortal database. Additionally, The Human Protein Atlas database was used to obtain the genotype-tissue expression (GTEx) dataset to examine the mRNA tissue expression of the genes in different tissues.

## Results

### WES analysis identified rare variants in candidate genes for cancer germline predisposition

In order to identify rare germline variants predisposing to familial cancers, including melanoma, in the Palestinian family, we performed WES on DNA samples obtained from a melanoma patient (II 9) and his two brothers, one diagnosed with a brain tumor (II 6) and the other with no medical history (II 5). The 36,521 VCF WES dataset of the three siblings was filtered (data available in Supplementary Table S1). A total of 3,838 uncommon, protein-affecting variants predicted to be pathogenic, with conflicting interpretation of pathogenicity and uncertain significance by ClinSig, were retrieved. Genes were further filtered based on run quality and genotype–phenotype correlation. This filtering reduced the number of variants to 33. Of these variants, 15 were previously associated with cancer and melanoma based on data of OMIM and ClinVar databases. These 12 genes included *ERCC2; KLC3, WRN, SMARCA4, SDHB, TYRP1, MRE11, TERT, MLH1, RELCH; PIGN, SNORD132; BUB1, MEN1, CDKN1A; DINOL, CDKN1A,* and *COMT; MIR4761*. Synonymous mutations in *COMT; MIR4761, CDKN1A*; *DINOL, RELCH; PIGN* genes were excluded as they did not affect splicing. Additionally, the exonic ncRNA mutations in the *SNORD132; BUB1* gene were excluded. The patient’s brother, who had a brain tumor, was treated as an affected family member. The top final variants and their corresponding genes most relevant to hereditary cancers and melanoma and present in both the patient and the sibling with a brain tumor, but not in the control, are in *WRN*, *TYRP1*, and *ERCC2*, shown in (Table 2) and discussed as follows.

**TABLE 2:** WES analysis and identification of germline candidate variants in the Palestinian family.

Gene	Mutation type	Amino acid change	ClinVar	Allele frequency (gnomAD)	II 9 genotype	II 5 genotype	II 6 genotype
*TYRP1*	Missense	c.C785T: p.T262M	Uncertain significance	0.0011	HET	-	HET
*ERCC2*	Missense	c. 2048G>A: p.R683Q	Pathogenic	0.0000159	HOM	HET	HOM
*WRN*	Missense	c.G1149T: p.L383F	Conflicting interpretations of pathogenicity	0.0021	HET	-	HET
*WRN*	Missense	c.G2983A: p.A995T	Conflicting interpretations of pathogenicity	0.0024	HET	-	HET

HET: heterozygote, HOM: homozygote.

All of the variants identified by WES analysis were missense mutations. The homozygous *ERCC2* mutation (NM_000400, c.2048 G>A, p.R683Q) was present in the patient and his brother with a brain tumor (6), while it is heterozygous in the unaffected brother (5). This mutation is classified as pathogenic in ClinVar. Moreover, it is associated with the phenotype xeroderma pigmentosum group D (XPD), characterized by cutaneous photosensitivity and predisposition to skin cancer on sun-exposed body sites, based on the OMIM description (OMIM #278730). The p.R683Q was observed in the gnomAD exome database with a low allele frequency of 0.0000159. In addition, it has been seen four times as a heterozygous mutation and never as a homozygous mutation (ID 19-45855609-C-T). The other missenses mutations in *TYRP1* (c.C785T, p.T262M) and *WRN* (c.G1149T: p.L383F, and c.G2983A: p.A995T) were present in a heterozygous state in the patient and his brother with a brain tumor (6), while it was absent in the unaffected brother (5). The *TYRP1* mutation is classified as unknown significance by multiple submitters in ClinVar. Homozygous or compound heterozygous mutations in TYRP1 are associated with oculocutaneous albinism type 3 (OCA3) (OMIM #203290), a milder form of OCA. In WRN, both of the mutations are classified as conflicting interpretations of pathogenicity based on ClinVar. They are present at an allele frequency of 0.0021 and 0.0024, respectively, in gnomAD and are associated with the autosomal recessive disorder, Werner syndrome (WRN) (OMIM #277700).

### The *ERCC2* missense mutation segregation in a Palestinian melanoma family

**FIGURE 1:**
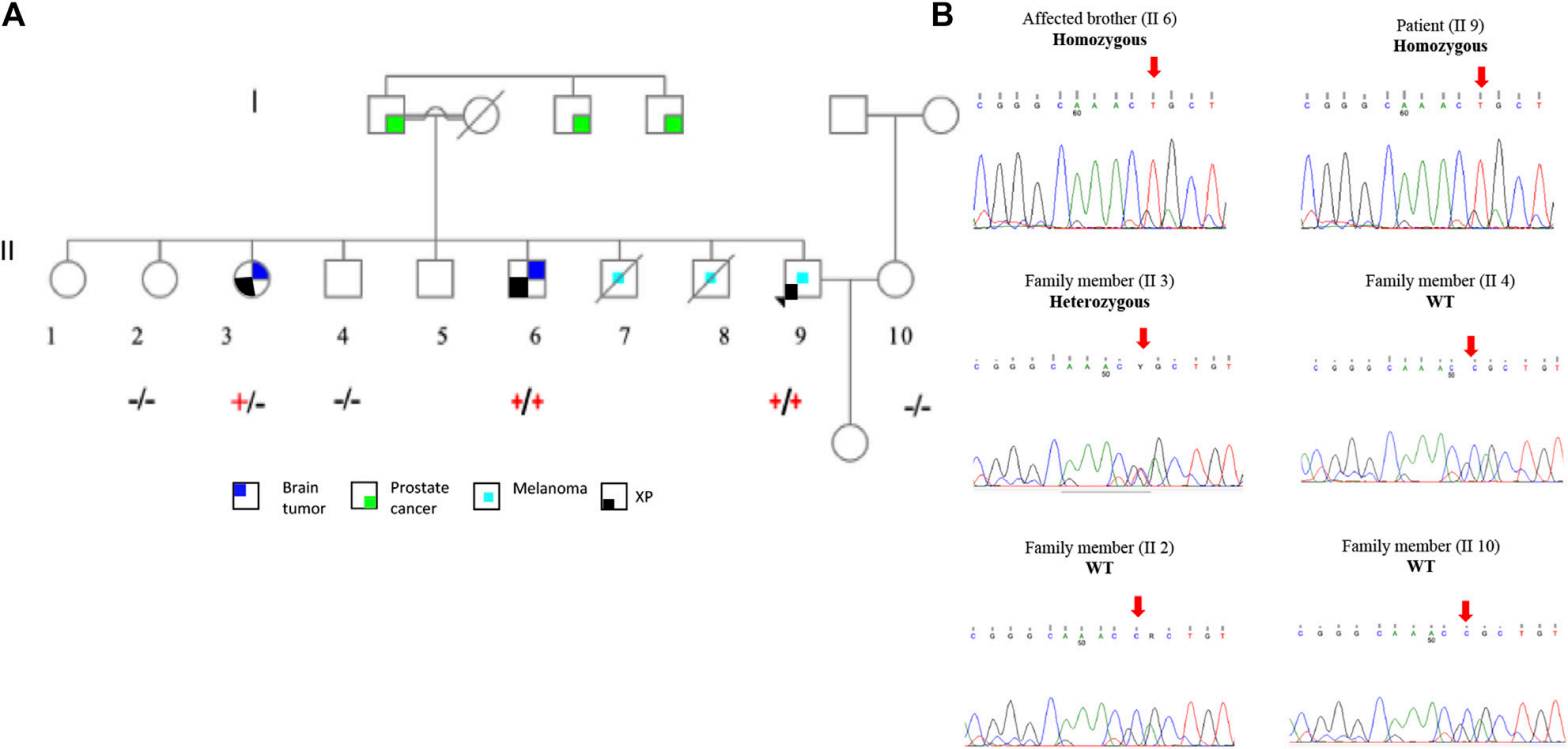
Family pedigree and Sanger sequencing segregation results of the p.R683Q mutation. **(A)**. Family pedigree showing the genotype of each member after Sanger sequencing analysis. The genotype +/+ (Homozygous), +/− (Heterozygous), or −/− (wild type) is represented below the symbol of each analyzed individual. **(B)**. Sequence chromatogram of the proband (II 9); affected brother (II 6); and family members II 2, II 3, II 4, and II 10. Red arrows show the mutation site.

To support the claim that p.R683Q mutation is the causative mutation in the tested family, we performed segregation analysis of the mutation between affected family members. The *ERCC2* missense mutation was selected for segregation analysis for the following reasons**:** 1) the p.R683Q mutation co-segregated with the disease phenotype in the patient and his affected brother (II 5) in a homozygous state and 2) classified as pathogenic in ClinVar. The *ERCC2* missense mutation was validated by Sanger sequencing, and co-segregation analysis was performed on the unaffected members in the family. The occurrence of homozygous missense mutation, p.R683Q, was confirmed in the patient (II 9) and his affected brother who has a brain tumor (II 6). Moreover, it was detected in a homozygous wild-type state in the patient’s brother (II 4), one sister (II 2), and his wife (II 10). It was also detected in a heterozygous state in the patient’s other sister with a brain tumor (II 3) (Figures 1A,B).

### *In silico* analysis of the *ERCC2* mutation and gene

**FIGURE 2:**
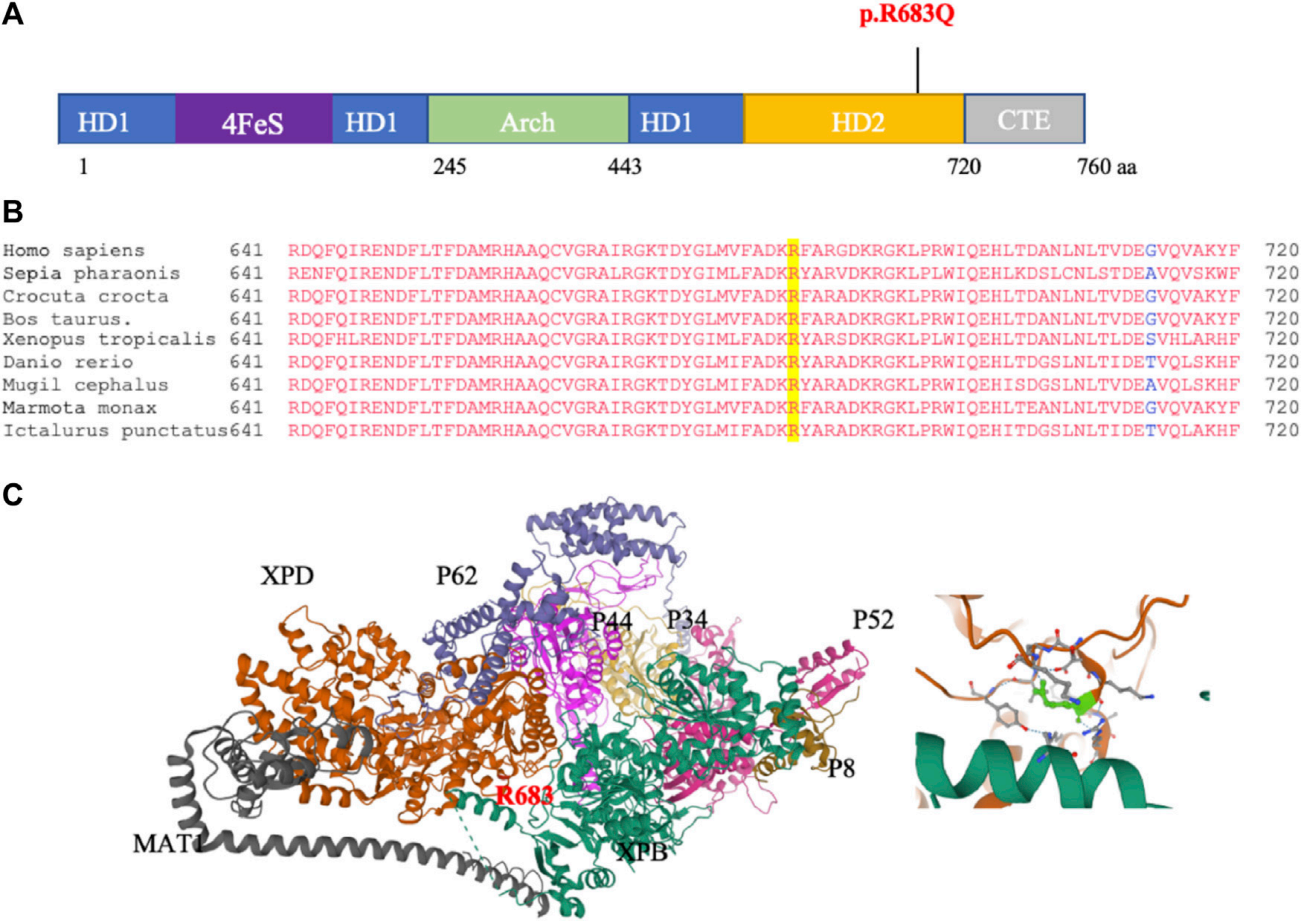
*In silico* analysis of p.R683Q pathogenicity. **(A)** Schematic of the ERCC2 domains and location of the mutation. The position of the p.R683Q mutation is identified in the helicase domain 2 (HD2) (indicated in red). **(B)** Multiple sequence alignment of the XPD protein regions surrounding the R683 (highlighted in yellow) that is mutated to p.R683Q. **(C)** Left: crystal structure of the (general transcription and DNA repair factor IIH helicase subunit XPD—chain B): (6NMI, PDB), showing the XPD and other subunits of the TFIIH, and the site of the R683 residue affected by the mutation (red). Right: zoomed-in view of the site of the mutation on the XPD structure (green).

To further confirm that the p.R683Q mutation is the disease-causing mutation in the tested family, we analyzed the possible pathogenicity of the mutation using *in silico* prediction tools. The schematic illustration of the ERCC2 protein (Figure 2A) shows that the p.R683Q is located in the ATP-dependent helicase region of the HD2 domain of the ERCC2 protein, required for the opening of the DNA. In addition, multiple alignment of amino acid sequences of the HD2 domain showed that residue 683 is highly conserved among different species (Figure 2B). In addition, the p.R683Q mutation is predicted with a high probability to be deleterious by all *in silico* algorithm tools (SIFT, PolyPhen2, Mutation Taster, Align GVD, and PROVEAN) (Table 3). Moreover, it is classified as pathogenic according to the ACMG guideline. The XPD 3D structure: (general transcription and DNA repair factor IIH helicase subunit XPD—chain B) was obtained from the Protein Data Bank (PDB) ID: 6NMI. The interaction of XPD with other subunits of the TFIH protein complex is characterized in this 3D structure. Moreover, it revealed the site of the mutation in the XPD protein structure (Figure 2C). To check whether or not the ERCC2 mutation is present in sequenced tumor samples of melanoma and other cancer types, it was carried out in COSMIC and cBioPortal databases. However, the mutation was not reported.

**TABLE 3:** Effect prediction of the melanoma candidate variant *ERCC2*. The variants were predicted by the *in silico* algorithms: SIFT (score under 0.05: “damaging”, score above 0.05; “tolerated”. Range 0–1), PolyPhen-2 (HumVar, “benign”-“possibly damaging”, “probably damaging”, Range: 0–1), PROVEAN (scores equal to or below −2.5 are considered “deleterious”, while scores above −2.5 are considered “neutral”. Align GVGD (scores (C0, C15, C25, C35, C45, C55, C65) from C0 “likely benign” to C65 “likely pathogenic”.

Gene	Mutation	SIFT	PolyPhen2	Mutation taster	Align GVD	PROVEAN
*ERCC2*	p.R683Q	Damaging (0)	Probably damaging (1)	Deleterious (0.992)	C35	Deleterious (−3.662)

**FIGURE 3:**
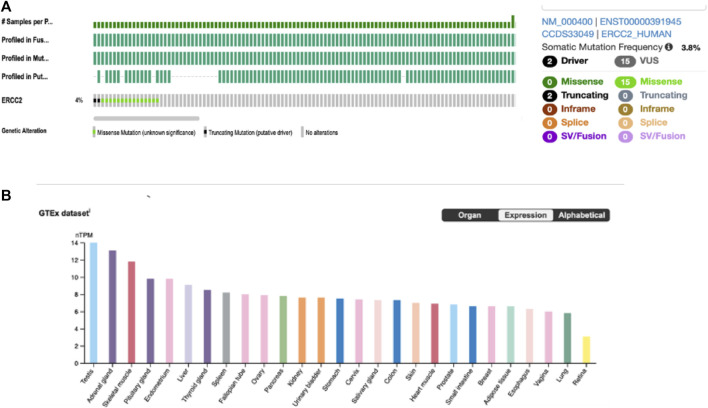
ERCC2 alteration in the online published data. **(A)** Alternation frequency (left) and types of mutations (right) of *ERCC2* in 448 cutaneous melanoma tumors from TCGA and PanCancer Atlas by the cBioPortal database. **(B)** RNA-seq analysis of human ERCC2 expression in different normal tissues obtained from the Human Protein Atlas, generated by the genotype-tissue expression database. Results are expressed by normalized transcript per million (nTPM).

Analysis of cBioPortal, the TCGA, and PanCancer Atlas database comprising 448 CM tumors found that ERCC2 is altered in 4% of the tumor samples (Figure 3A, left). In addition, 3.8% of the alternations were somatic mutations (n = 17) (Figure 3A, right), of which two were truncated (drivers) and the rest were missense mutations of uncertain significance forming the predominant type of the mutation. This demonstrates that melanoma genetics is complex and that there are likely many mutations that need to be studied to find out the driver mutations causing disease development and progression. We then used the genotype-tissue expression (GTEx) to evaluate the mRNA expression of the ERCC2 in different tissues. The *ERCC2* was expressed in numerous tissues with similar values, including those of the testis, prostate, skin, and endometrium (Figure 3B).

## Discussion

In this study, we used WES technology to identify rare germline variants predisposing to hereditary cancer in a Palestinian family. Analysis of the WES data of the proband and his two siblings revealed four rare variants in *WRN, TYRP1*, and *ERCC2* candidate genes. Our study identified a pathogenic missense mutation in *ERCC2* (rs758439420) on chromosome 19, leading to a substitution of arginine by a glutamine at codon 683 (p.R683Q). *ERCC2* encodes an ATP-dependent DNA helicase called XPD that is important in the DNA damage repair by the NER mechanism (B et al., 2018). According to GTEx, the mRNA of ERCC2 shows a high expression in numerous tissues, suggesting the essential function of *ERCC2* in maintaining genome integrity through the NER pathway. Thus, mutations in this gene are associated with the susceptibility to various cancer types including bladder cancer, lung cancer, melanoma, and colon cancer (RAD50 - My Cancer Genome, 2023; Van Allen et al., 2014; Liu et al., 2023; Zhang et al., 2022; Díaz-Gay et al., 2019). However, *ERCC2* mutations seem to be uncommon in CM tumors since only 4% of cases from the TCGA data harbored an alteration in *ERCC2*, and specifically two truncating mutations were drivers. The p.R683Q mutation was absent from the COSMIC and cBioPortal databases, indicating that this mutation in the *ERCC2* gene cannot be a somatic mutation. Germline mutations in *ERCC2* have been rarely found associated with hereditary cancer. Biallelic germline mutations in ERCC2 are associated with the rare autosomal recessive disorder, xeroderma pigmentosum group D (XPD). XP patients with mutations in *ERCC2* are characterized by photosensitivity; abnormality in skin pigmentations in the UV-exposed area of the body; neurodegeneration; and an increased risk of developing skin cancers, brain tumors, and tumors in other organs (B et al., 2018; Black, 2016). The p.R683Q missense mutation was present only four times at a heterozygous state and never at a homozygous state in the gnomAD database. This indicates the rarity of the alleles and evidence for pathogenicity. In our study, *in silico* analysis of the 3D structure—general transcription and DNA repair factor IIH helicase subunit XPD— showed the different subunits of the TFIH protein complex, and identified the site of the mutation, p.R683Q, in XPD. The p.R683Q mutation is found toward the C-terminal end of the protein. Most mutations in XPD patients, including ours are within codon 683, among whom the Arg683Trp is the most predominant, (Moura and Houten, 2023) reported in more than 80% of the patients (Santiago et al., 2020). Functional studies have shown that mutations at the C-terminal end of the protein interfere with the p44 binding, thus preventing the stimulation of the helicase activity of XPD (Coin et al., 1998). R683 is also involved in binding to double-strand/single-strand DNA junctions, and as expected, replacing R residue with Q at position 683 changes the positive charge, resulting in diminished DNA binding. Therefore, this explains the deficit in the NER system observed in XP patients (Bui et al., 2020). A difference in clinical phenotypes has been reported, with the phenotype in patients with Arg683Trp being more severe than the phenotype observed in patients with Arg683Gln (B et al., 2018). Previous *in silico* analysis has found that Arg683Gln has more ATPase activity than the Arg683Trp. The *ERCC2* pathogenic missense mutation identified by our study (c. 2048G>A, p.R683Q) was present in a homozygous state in the patient, who was diagnosed with melanoma at the age of 30 (Nakano et al., 2014). In addition to melanoma (HP:0002861), the patient presented with an abnormality of skin pigmentation and hyperpigmentation of the skin (HP:0001000; HP:0000953). Notably, the homozygous variant was also identified in the patient’s brother (6), who had a brain tumor besides the same clinical phenotypes found in the patient, except for melanoma. These findings correlate with the phenotypic features of XPD, including the high risk of developing CM and other cancer types, as a result of exposure to ultraviolet light from the Sun. Furthermore, WES results showed that the unaffected individual (5) is heterozygous for the variant. The patient’s parents must also be considered heterozygous, implying that *ERCC2* has an autosomal recessive inheritance in familial cases. To confirm that p.R683Q mutation is the mutation that predisposes the family to different cancers, we conducted Sanger sequencing of the area flanking the mutation site. Sanger sequencing analysis has confirmed the homozygous mutation and has shown that one of the unaffected family members is heterozygous, while the three remaining members were wild-type (WT). The patient’s daughter should be heterozygous since her mother was tested to be WT for the mutation. Individuals (5) and (3) who are known to be carriers have a high risk of transmitting the mutation to the next generation. These findings confirm that the homozygous *ERCC2* variant (p.R683Q) is absent in non-affected family members. Moreover, it co-segregated with the phenotype in the investigated patient and in his brother with a brain tumor. Therefore, it is the mutation responsible for predisposing the studied Palestinian family to hereditary cancer and melanoma. The Arg683Gln mutation has been previously reported at a homozygous state in patients from various ethnic backgrounds, including Italian, German, Iraqi Jewish, Japanese, and Tunisian (B et al., 2018; Nakano et al., 2014; Taylor et al., 1997). The study on Tunisian families has identified ten homozygous patients belonging to three families and has shown full co-segregation. These patients exhibited mild dermatological manifestations, late onset of skin tumors, and an absence of neurological abnormalities (B et al., 2018). A recent study in Vietnam has reported that four siblings affected by XP with extreme Sun sensitivity carried compound heterozygous mutations in *ERCC2*, p.R683Q in one allele of the gene and a novel p.Q452X nonsense mutation in the other allele. The study suggested that the latter mutation is responsible for the severe Sun sensitivity experienced by the XP patients in contrast to the p.R683Q mutation that is associated with mild Sun sensitivity (Bui et al., 2020). This indicates that the different XPD mutations strongly influence the range and severity of the phenotypes. The study has shown that two of the patients were diagnosed at the ages of 38 and 35 and had developed melanoma. Additionally, they experienced phenotypes such as hyperpigmentation, irritation, and freckles (Bui et al., 2020). The patients in our study were not diagnosed previously with XP, and this is because they presented with mild clinical phenotype and late onset of clinical manifestations. Hence, the lack of awareness and knowledge about this syndrome played a role in the progression of the disease. We think that the identified homozygous *ERCC2* p.R683Q mutation could have contributed to the tumorigenesis in the patient’s brother (36 years old), who has a brain tumor. Most probably, the brain tumor resulted from an increased rate of mutations due to the defective DNA repair caused by the *ERCC2* mutation. This assumption is based on the fact that the p.R683Q mutation falls in the helicase domain of the ERCC2 protein, and in fact it was shown before that mutations in the helicase domain of ERCC2 confer NER deficiency (Li et al., 2019). In fact, the brother with the brain tumor is still at a high risk of developing melanoma, due to the strong history of melanoma in the family. Moreover, reports have suggested that the age of developing melanoma in patients with XP ranges between 2 and 47 years (Bradford et al., 2011). On the other hand, carriers of the *ERCC2* p.R683Q do not have a risk of developing melanoma. Even though haploinsufficiency could play a role in increasing the susceptibility to cancer without leading to a cancer syndrome, there have been no formal documents attributing cancer susceptibility to a heterozygous variant in a gene of an autosomal recessive cancer syndrome. Moreover, loss of heterozygosity (LOH) is not a common event in patients with *ERCC2* mutations. Only one study has reported the presence of LOH in tumors from patients with *ERCC2* germline mutations, and this has been found in bladder cancer patients carrying the p.Arg616Pro mutation in *ERCC2* (Carlo et al., 2020). A limitation of this study is that the patient’s parents refused to participate. However, we were able to include siblings and perform a segregation analysis. Finally, it is important to mention that functional studies are needed to ensure a causation relationship between *ERCC2* p.R683Q mutation and the cancer syndrome in the family.

## Conclusion

In summary, we have successfully applied whole-exome sequencing for an unexplained molecular cause of melanoma for diagnosis of XP and identified a rare *ERCC2* missense mutation (c. 2048G>A, p.R683Q) responsible for the development of melanoma and other cancer types in the family. This study expands the knowledge of the mutational background of familial cancers, including melanoma, in the Palestinian population, which is valuable in guiding the diagnosis, prevention, and treatment of affected patients and their families.

## Data Availability

The original contributions presented in the study are publicly available. This data can be found here: https://www.ncbi.nlm.nih.gov/bioproject/1030526.
